# Allocation of Study Time in Chinese Junior School Students: Habitual Responding, Item Difficulty, and Time Constraints

**DOI:** 10.3389/fpsyg.2016.00639

**Published:** 2016-05-03

**Authors:** Fuyun Wang, Qiwen Qin, Yanju Jiang

**Affiliations:** ^1^Southwest UniversityChongqing, China; ^2^Henan UniversityKaifeng, China

**Keywords:** study time allocation, junior school students, habitual responding, agenda-based regulation

## Abstract

The present study investigated factors influencing Chinese junior school students’ study time allocation and the age difference in the effect of habitual responding. Participants were 240 junior school students (120 girls, 120 boys; aged 13–15 years) with half taking part in Experiment 1 and half in Experiment 2, and 240 young adults aged 18–23 years, (120 women and 120 men,) involved in Experiments 3a and 3b, all native Chinese speakers. In Experiments 1 and 3a, Chinese word pairs (e.g., moon–star) were presented on the screen with three items in one array. In each trial, the items were arranged from left to right, either easy, moderate, then difficult, or the reverse. Participants had either 5 s or no time limits to study the word pairs. In Experiments 2 and 3b, word pairs were ordered in a column with the easiest items either at the top or bottom position. Results showed interactions among item difficulty, item order, and time limitation in terms of effects on study time allocation of junior school students. Participants tended to learn the items in order (from left to right and from top to bottom), but the effect of item difficulty was greater than that of item order on item selection. Results indicated that agenda and habitual responding have a combined effect on study time allocation and that the contribution of agenda is greater than that of habitual responding. The effect of habitual responding on the self-paced study and recall performance of junior school students is greater than its effect on young adults, and the study time allocation of junior school students is more likely to be affected by external conditions.

## Introduction

Study time allocation is a core aspect of metacognitive control. It refers to the process whereby learners allocate their own subjective effort and attention, and it reflects the individual’s ability to understand the learning task and to choose how to engage with it (Perfect and Schwartz, 2002). It is an important part of self-regulated study, and how people allocate learning time has been the focus of research on study time allocation. Many empirical studies have revealed factors that influence the process and results of study time allocation, such as habitual responding (Metcalfe, 2002; Dunlosky and Ariel, 2011b), item difficulty (Metcalfe and Kornell, 2005; Pyc and Dunlosky, 2010; Ariel and Dunlosky, 2013), and time constraints (Metcalfe and Kornell, 2003).

### The Model of Study Time Allocation

People use metacognition to guide their study behaviors and control over their own future learning; it enables them to allocate their study time effectively (Nelson et al., 1994; Metcalfe and Kornell, 2003). Executive control of metacognition includes selecting stimulus information, maintaining information in working memory, and manipulating information processing (Shimamura, 2000). In order to explain how learners allocate their study time, a number of theories based on metacognition have been proposed, such as the region of proximal learning (RPL) framework (Metcalfe, 2002) and the agenda-based regulation (ABR) framework (Thiede and Dunlosky, 1999). Son and Metcalfe (2000) found that time pressure can affect learners’ study time allocation. When people are given a very short amount of time, they tend to spend more time on easy items than on difficult items in order to achieve good test results. To explain this phenomenon, Metcalfe (2002) put forward the RPL framework. According to the RPL, people always select the easiest items from those they have yet to learn. This is particularly, so if learners do not have enough time and ability to master the most difficult items; in this situation, they tend to defer their study of the difficult items in order to review the easy items.

The present study utilizes the ABR framework as the model of study-time allocation (Thiede and Dunlosky, 1999). According to the ABR framework, ‘study-time allocation will be driven by multiple sources: agenda construction, agenda execution, habitual responding, and online monitoring and control’ (Dunlosky and Ariel, 2011a, p. 122). In this model, agenda construction is the first step in study time allocation. Learners build an agenda to plan how to spend time on different items, and then make study decisions based on this agenda (Benjamin, 2008). Agenda construction is goal-directed, with the aim of maximizing the possibility of achieving the goals effectively. Habitual responding and meta-cognitive monitoring also affect the construction and execution of the agenda. Habitual responding is an individual behavioral response that is stimulated by a combination of environmental stimuli and the individual’s previous experience (Dunlosky and Ariel, 2011b). Depending on habitual responding, the order of items can affect learners’ agendas (Ariel et al., 2011). If the goal is to master all the materials, learners always start from the beginning and select items according to the item order. If the goal is to master only some of the materials, learners may build an agenda according to this goal (Ariel et al., 2009).

Agenda-based processes are compatible with the RPL theory, because according to the RPL model, learners usually choose the easiest unlearned items to study first. However, the ABR framework proposes that, in addition to the difficulty of study materials, learners’ study decisions are influenced by habitual responding (Dunlosky and Ariel, 2011b). For example, when learners study unconsciously, they always choose items according to habitual responding rather than using more effective methods to achieve learning goals. Ariel et al. (2009) point out that:

Even so, the ABR framework predicts that habitual responses will influence study-time allocation and can undermine ABR, and these habitual responses are expected to have a larger influence when the capacity of the central executive is exceeded. In this case, learners are expected to forget their agenda or learning goal and revert to habitual responding (p. 124).

### The Influence of Study Time Allocation Factors

Most studies have identified item difficulty as one of the most important factors affecting study time allocation (Nelson and Leonesio, 1988; Singell and Lillydahl, 1996; Ariel et al., 2009; Weinstein and Roediger, 2010; Ariel and Dunlosky, 2013). Researchers have used different methods to manipulate the difficulty of experimental learning items, such as word length (Belmont and Butterfield, 1971), the degree of word association (Niu et al., 2010), familiarity with the material (Kobasigawa and Metcalf-Haggert, 1993), and the learning rate of the material (Metcalfe and Kornell, 2005). Most research has used words as learning materials, but some researchers have also used pictures (Kobasigawa and Metcalf-Haggert, 1993). Son and Metcalfe’s (2000) review summarized all the published literature on the relation between difficulty and study time, examining 46 treatment combinations. The results showed that in 80% of studies, participants spent the most time on difficult items, and in 2% of studies, participants spent the most time on moderately difficult items. In 18% of studies, there were no significant differences in time spent on materials of varying levels of difficulty; however, these results were drawn from either teenagers with intellectual difficulties (Belmont and Butterfield, 1971) or infants (Kobasigawa and Metcalf-Haggert, 1993).

Time constraint also has a significant effect on study time allocation. Dunlosky and Ariel (2011b) found that when people were given a very short amount of time to learn materials, they tended to study the easy items first, and the effects of item order were weakened. When people are not under time constraints, they tend to study the items in order, and the effects of item difficulty on item selection are weaker. Some Chinese researchers (Liu and Fang, 2006a) have used time limits (short, medium, and unlimited time) as an independent variable to investigate the developmental characteristics of primary school students’ self-paced study time. The results showed that for short time limits, there were no significant differences in the time Grades 4 and 5 students spent on items of different levels of difficulty. For medium time limits, there was a significant difference in the time spent on easy and difficult items, but no significant difference between moderately difficult items and difficult items. When there was no limit on time, there were significant differences in the time spent on items of different levels of difficulty in self-paced study time. Niu et al. (2010) found that having a time limit restricts the effect of score on time allocation. When there is no time limit, the effect of score is weaker than for short time limits.

The habitual responding had many influences on people’s preferences, such as decision making, attentional momentum, and the way they represent numbers and time (e.g., Chokron and De Agostini, 2000; Spalek and Hammad, 2005; Shaki et al., 2009). Metcalfe (2002) and Ariel et al. (2011) found that habitual responding could influence study time allocation. In Metcalfe’s paradigm, three words are presented on the computer screen at the same time, divided into three different difficulty levels (easy, medium, and difficult). Since item order is one of the variables, items are ordered from left to right with either the easiest or the most difficult items on the left. Results show that when difficult items are on the left, participants tend to choose these items, rather than the easier items, to study first. Dunlosky and Ariel (2011b) speculated that this is due to reading habit. In order to verify this speculation, they studied native Arabic speakers, whose habitual responding are from right to left, using the same procedures as Metcalfe (2002). As predicted, the participants tended to choose items from right to left.

Research indicates that participants of different ages allocate their study time differently (Flavell, 1971; Liu and Fang, 2006a,b). Adults tend to opt to study difficult items first, and the allocation of study time varies according to the difficulty of items. However, Grade 1 primary school students do not significantly vary study time according to item difficulty, and the selection order of study items is random. Grade 3 primary school students begin to allocate study time on the basis of item difficulty, but the variation is less than that of adults (Liu and Fang, 2006b). Research on adolescents and elderly people shows that the most time is allocated to difficult items (Froger et al., 2012). Age difference also affects the components of metacognition, which can affect study time allocation. Research on working memory across the life span showed that young adults had higher working memory capacity and greater ability to inhibit irrelevant information than junior school students had (Swanson, 1999; Zhang and Liu, 2006).

### The Present Study

Most recent studies have focused on study time allocation in children or adults, with little studies of allocation of study time in junior school students. The present study aimed to investigate how Chinese junior school students allocate learning time and the nature of their study time allocation. The effect of habitual responding has been examined in both native English and Arabic speakers (Ariel et al., 2011; Ariel and Dunlosky, 2013), but the research on native Chinese speakers is insufficient. We therefore aimed to investigate whether habitual responding could influence the study time allocation of learners who have different native languages. Two specific hypotheses were tested. First, that habitual responding, item difficulty, and time constraints would have a combined effect on the study time allocation of junior school students. They would tend to choose study items according to habitual responding and would first study items in the most prominent (leftmost or topmost) position of an array. However, when the difficult items were presented in the most prominent position, we predicted that this tendency would be weaker. When the time constraint was long, item order would have little effect on item selection, and junior school students would tend to select items according to habitual responding. Second, we predicted that the effect of habitual responding on junior school students would be greater than on adults.

## Experiment 1

### Method

#### Participants

Participants were 120 students (50% boys and 50% girls; mean age = 13.87 years, *SD* = 0.62), randomly selected from Grade 2 of one junior school in Zhengzhou, Henan province. Their vision or corrected visual acuity was normal, and they were all right-handed. Participants volunteered for the experiment, and none had previously taken part in similar experiments. All participants received a small gift (a colored cover note book) at the end of the experiment.

#### Materials

A total of 96 Chinese word pairs adapted from Niu (2006, unpublished) were used. The word pairs were divided into 32 easy pairs (e.g., moon–star), 32 moderately difficult pairs (e.g., pen–school), and 32 difficult pairs (e.g., channel–fan) according to the degree of association.

#### Design

The experiment was a 3 (Item Difficulty: easy, moderately difficult, difficult) × 2 (Item Order: easy on the left [EMD] vs. difficult on the left [DME]) × 2 (Time Allowed: 5 s vs. unlimited) mixed factorial design. Item difficulty and time allowed were within-subject factors. Participants were randomly assigned to either the EMD group (*n* = 30) or the DME group (*n* = 30). We chose 5 s as the short time limit condition, following Dunlosky’s methods.

#### Procedure

The experimental materials and tests were presented on a computer screen (the word pairs were white and the background was black). Participants were informed that they would be asked to learn 96 associated word pairs. They were instructed that on each trial, three target words would be presented on the computer screen and the difficulty (easy, moderately difficult, or difficult) was presented above the word. When participants clicked on the question mark button below the word (the word would remain on the screen until they clicked on another question mark or the study time elapsed) the other word of the pair would be displayed. In the EMD group, the participants were informed that the item order from left to right was easy items, moderately difficult items, and difficult items on each trial. In the DME group, the participants were informed that the easy word pairs were on the right and the difficult word pairs were on the far left.

The 96 word pairs were divided into two timing conditions, with 16 trials (three word pairs in one trial, a total of 48 word pairs) in each condition. In one condition, participants were given 5 s to study and select the words; in the other condition, they had no time limits, In the 5-s condition, the computer skipped to the next trial automatically when the time elapsed. In the unlimited time condition, participants could click the button labeled ‘next trial’ on the screen to move to a new study trial. At the end of each condition, participants were tested on each word pair. During testing, the target word was presented and participants had to type the other word of the word pair. The order of the words presented in the post-test was random. After completing the test in the time allowed, participants had 5 min to rest before they attempted the other time condition. The order of the timing conditions (5 s first vs. unlimited first) was random.

### Results and Discussion

The data were coded for analysis with the Statistical Package for the Social Sciences (SPSS) 18.0, and a cut-off value of *p* < 0.05 was chosen as the criterion for significance. Simple effect analysis was used when interaction terms were significant. The order of item selection, self-paced study times, and recall performance were the dependent variables.

#### Item Selection

**FIGURE 1:**
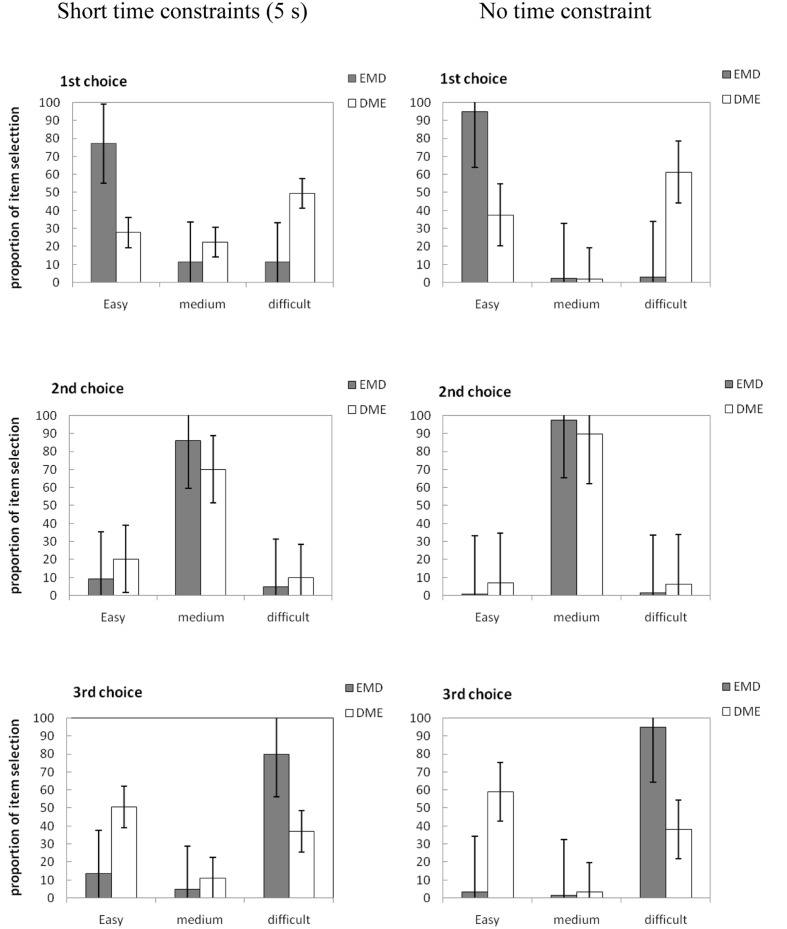
**Mean proportion of items selected for the first, second, and third choice across the word pair arrays in Experiment 1.** E, easy word pair; M, moderately difficult word pair; D, difficult word pair. Error bars are standard errors of each mean.

We calculated the percentage of times out of 16 trials that items of each difficulty level (easy, moderately difficult, or difficult) were selected first, second, and third. Participants’ mean selection percentages for items of different difficulty are presented in **Figure 1.**

The results show that for the short time limit (5 s), participants selected 77.18% of easy items first in EMD (easy items on the left) and selected 49.48% of difficult items first in DME (difficult items on the left). Participants selected 80% of difficult items third in EMD (difficult items on the right) and selected 50.42% of easy items third in DME (easy items on the right). The largest percentage of participants’ second choice for the two groups (EMD, DME) was for moderately difficult items (85.94 and 70.10%, respectively). The participants also tended to choose items from left to right when they had no time limit.

Dunlosky and Ariel (2011b, p. 903) suggests that:

…the three choices (and choices among the three levels of item difficulty) are not statistically independent; once the proportion of item selection is known for two of the three choices (or for two of the three levels of item difficulty), the other value is fixed. Thus, our analyses excluded the second choice and the moderately difficult items.

We therefore retained just two choices (first and third), and conducted a separate 2 (Item Order: EMD vs. DME) × 2 (Time Limit: 5 s vs. unlimited) × 2 (Item Difficulty: easy or difficult) analysis of variance (ANOVA).

The ANOVA for first choices revealed a significant main effect of item difficulty, *F*(1,118) = 83.58, $\eta_{p}^{2}$ = 0.42, *p* < 0.01. Item difficulty interacted with item order, *F*(1,118) = 12.97, $\eta_{p}^{2}$ = 0.10, *p* < 0.01. The simple effect analysis showed that the item order was different for easy items, *F*(1,118) = 262.33, *MSE* = 12.13, *p* < 0.01, but not for difficult items, *F*(1,118) = 1.34, *MSE* = 0.34, *p* = 0.25. The descriptive statistics showed that when the easy items were presented on the left, they were prioritized during selection (EMD, *M* = 85.94%, *SD* = 11.03%), compared with when they were presented on the right (DME, *M* = 32.60%, *SD* = 20.66%), *t*(60) = 17.64, indicating that item order affected the selection of easy items. However, easy items were prioritized during selection when they were presented on the left (EMD, *M* = 85.94%) compared with difficult items presented in the left position (DME, *M* = 55.36%), *t* = 10.52, indicating that the effect of item difficulty on study selection is greater than item order. The main effect of time allowed was significant, *F*(1,118) = 98.90, $\eta_{p}^{2}$ = 0.46, *p* < 0.01. Time allowed interacted with item order, *F*(1,118) = 325.10, $\eta_{p}^{2}$ = 0.73, *p* < 0.01. The simple effect analysis showed that the time allowed was different for both the EMD, *F*(1,118) = 311.16, *MSE* = 4369.07, *p* < 0.01, and the DME conditions*, F*(1,118) = 288.46, *MSE* = 3565.10, *p* < 0.01, indicating that the time allowed affected the order of study choice, and that junior school students preferred to choose items according to item order when there was no time limit, compared to when the time limit was short (5 s).

An ANOVA of third choices revealed a significant main effect of item difficulty, *F*(1,118) = 73.71, $\eta_{p}^{2}$ = 0.24, *p* < 0.01. There is a marginal significant interaction effect between item difficulty and item order, *F*(1,118) = 3.76, $\eta_{p}^{2}$ = 0.03, *p* = 0.05. The simple effect analysis showed that item order was different for easy items, *F*(1,118) = 7.00, *MSE* = 16.02, *p* < 0.01, but not for difficult items, indicating that the effect of item order on easy items is greater than on difficult items. The descriptive statistics indicated that as their third choice, participants preferred selecting difficult items (*M* = 62.5%) compared with easy items (*M* = 31.16%) when either was in the far right position. The main effect of time allowed was significant, *F*(1,118) = 120.05, $\eta_{p}^{2}$ = 0.50, *p* < 0.01. Time allowed interacted with item difficulty, *F*(1,118) = 291.83, $\eta_{p}^{2}$ = 0.69, *p* < 0.01. The simple effect analysis showed that time allowed was different for easy items, *F*(1,118) = 36.57, *MSE* = 1066.82, *p* < 0.01; for difficult items, a main effect was found for time allowed, *F*(1,118) = 62.08, *MSE* = 1915.35, *p* < 0.01.

#### Self-paced Study

Mean self-paced study times are presented in **Table 1.**

**Table 1:** Self-paced study time in seconds for Experiment 1 (Ms, SDs).

Item order	Short time constraints (5 s)				No time constraint		
			
	Easy	Moderate	Difficult		Easy	Moderate	Difficult
EMD	1.33	1.50	1.54		4.87	7.87	9.60
	*SD* = 0.21	*SD* = 0.25	*SD* = 0.25		*SD* = 1.77	*SD* = 3.28	*SD* = 5.14
DME	1.27	1.87	1.43		8.39	16.36	14.43
	*SD* = 0.19	*SD* = 0.4	*SD* = 0.19		*SD* = 2.53	*SD* = 4.37	*SD* = 2.86


*EMD, easy items on the left of the array and difficult items on the right; DME, difficult items on the left of the array and easy items on the right.*

An ANOVA revealed a significant main effect of item difficulty, *F*(1,118) = 172.32, $\eta_{p}^{2}$ = 0.75, *p* < 0.01, a significant main effect of time allowed, *F*(1,118) = 1219.44, $\eta_{p}^{2}$ = 0.91, *p* < 0.01, and a significant main effect of item order, *F*(1,118) = 130.99, $\eta_{p}^{2}$ = 0.53, *p* < 0.01. Item difficulty interacted with item order, *F*(2,117) = 33.00, $\eta_{p}^{2}$ = 0.36, *p* < 0.01. The simple effect analysis showed that item order was different for both easy items, *F*(1,118) = 22.32, *MSE* = 1.48, *p* < 0.01, and difficult items, *F*(1,118) = 106.44, *MSE* = 2666.99, *p* < 0.01, indicating that item order affects self-paced study. The descriptive statistics showed that participants spent more time on DME (*M* = 7.29) than on EMD (*M* = 4.36). Time allowed interacted with item order, *F*(1,118) = 122.17, $\eta_{p}^{2}$ = 0.51, *p* < 0.01. The simple effect analysis showed that the time allowed was different for both the short time constraint condition, *F*(1,118) = 693.12, *MSE* = 142.27, *p* < 0.01, and the unlimited conditions*, F*(1,118) = 77.72, *MSE* = 762.18, *p* < 0.01, indicating that the item order affected the time which spent on the different difficulty. Time allowed interacted with item difficulty, *F*(1,118) = 139.39, $\eta_{p}^{2}$ = 0.70, *p* < 0.01. The simple effect analysis showed that time allowed was different for both the short time limit (5 s), *F*(2,236) = 904.78, *MSE* = 4601.37, *p* < 0.01, and no time constraint, *F*(1,118) = 515.65, *MSE* = 3202.65, *p* < 0.01.

#### Recall Performance

We examined recall performance to determine the effect of study time allocation. The mean test accuracies are presented in **Table 2.**

**Table 2:** Test accuracies for Experiment 1 (Ms, SDs).

Item order	Short time constraints (5 s)				No time constraint		
			
	Easy	Moderate	Difficult		Easy	Moderate	Difficult
EMD	0.36	0.19	0.05		0.66	0.54	0.26
	*SD* = 0.07	*SD* = 0.10	*SD* = 0.10		*SD* = 0.16	*SD* = 0.24	*SD* = 0.23
DME	0.32	0.25	0.05		0.69	0.55	0.20
	*SD* = 0.06	*SD* = 0.14	*SD* = 0.12		*SD* = 0.19	*SD* = 0.17	*SD* = 0.17


*EMD, easy items in the left of the array and difficult items in the right; DME, difficult items in the left of the array and easy items in the right.*

An ANOVA revealed a significant main effect of item difficulty, *F*(2,117) = 628.25, $\eta_{p}^{2}$ = 0.92, *p* < 0.01, and a significant main effect of time allowed, *F*(1,118) = 333.01, $\eta_{p}^{2}$ = 0.74, *p* < 0.01, but no significant main effect of item order. Item difficulty interacted with item order, *F*(2,117) = 4.13, $\eta_{p}^{2}$ = 0.07, *p* < 0.05. The simple effect analysis showed that the item difficulty was different for both the EMD, *F*(1,118) = 23.37, *MSE* = 34.84, *p* < 0.01, and the DME conditions*, F*(1,118) = 12.01, *MSE* = 17.91, *p* < 0.01, indicating that the item order affected the recall performance of junior school students on different difficulty of item. Time allowed interacted with item difficulty, *F*(1,118) = 163.24, $\eta_{p}^{2}$ = 0.58, *p* < 0.01. The results indicated that item difficulty and time constraint jointly affect recall performance, and that item order has no effect on recall performance.

## Experiment 2

### Method

In Experiment 1, we investigated the influences of habitual responding, item difficulty, and time constraint on study time allocation when the materials were structured horizontally (from left to right). In Chinese, writing and reading order are not only from left to right, but also from top to bottom. In order to verify the effect of habitual responding and agenda, based on the RPL, we presented the material in a longitudinal structure (from top to bottom).

#### Participants

Participants were 120 students (50% boys and 50% girls; mean age = 14.12 years, *SD* = 0.79), randomly selected from Grade 2 of one junior school in Zhengzhou, Henan province.

#### Materials and Procedure

The materials used were the same as for Experiment 1. The procedure followed that of Experiment 1, with the exception that the study material was presented vertically instead of horizontally. In the EMD group, the item order from top to bottom was easy items, moderately difficult items, and difficult items for each trial. In the DME group, easy word pairs were presented at the bottom, and difficult word pairs were presented at the top.

### Results and Discussion

#### Item Selection

**FIGURE 2:**
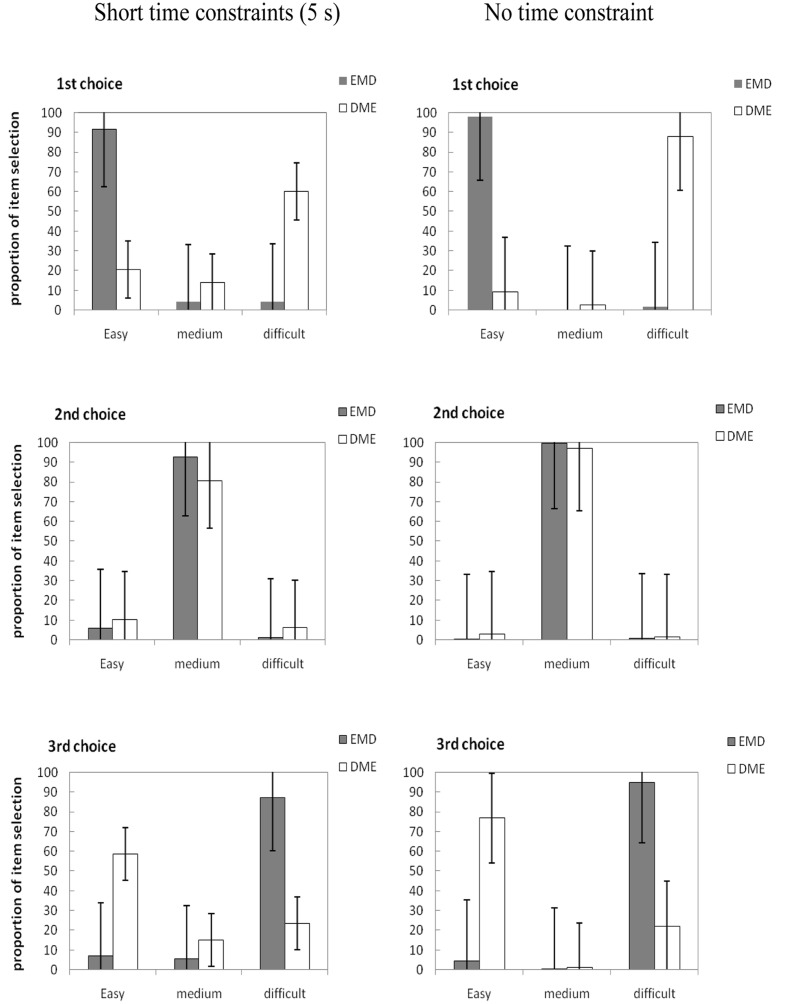
**Mean proportion of items selected for the first, second, and third choice across the word pair arrays in Experiment 2.** E, easy word pair; M, moderately difficult word pair; D, difficult word pair. Error bars are standard errors of each mean.

We calculated the percentage of times out of 16 trials that items of each difficulty level (easy, moderately difficult, or difficult) were selected first, second, and third. Participants’ mean selection percentages for items of different difficulty are presented in **Figure 2.**

**Figure 2** shows that for the short time limit (5 s), participants were most likely to select easy items first in the EMD group (91.56%); for the third choice, difficult items were more likely to be selected (87.18%). In the DME group, participants were most likely to select difficult items first (60%); for the third choice, easy items were more likely to be selected (58.64%). The largest percentage of participants’ second choice for the two groups (EMD, DME) was moderately difficult items (92.6 and 80.62%, respectively). Participants tended to choose items from top to bottom when there was no time constraint.

As in Experiment 1, we retained just the first and third choices and conducted a separate 2 (Item Order: EMD vs. DME) × 2 (Time limit: 5 s vs. unlimited) × 2 (Item Difficulty: easy or difficult). The ANOVA for first choices revealed a significant main effect of item difficulty, *F*(1,118) = 36.06, $\eta_{p}^{2}$ = 0.23, *p* < 0.01, and a significant main effect of time allowed, *F*(1,118) = 35.39, $\eta_{p}^{2}$ = 0.23, *p* < 0.01. The main effect of item order was also significant, *F*(1,118) = 14.31, $\eta_{p}^{2}$ = 0.11, *p* < 0.01. Item difficulty interacted with item order, *F*(1,118) = 760.95, $\eta_{p}^{2}$ = 0.87, *p* < 0.01. The simple effect analysis showed that item order was different for both easy items, *F*(1,118) = 14.55, *MSE* = 88.82, *p* < 0.01, and difficult items, *F*(1,118) = 5.86, *MSE* = 2.4, *p* < 0.05. The descriptive statistics showed that when the easy items were presented on the top, they were prioritized during selection (EMD, *M* = 94.79%, *SD* = 6.94%) compared with when they were presented on the bottom (DME, *M* = 15.00%, *SD* = 19.31%), *t*(60) = 30.11, indicating that item order affects the order of study. However, easy items were prioritized during selection when they were presented on the top (EMD, *M* = 94.79%, *SD* = 6.94%), compared with difficult items presented in the top position (DME, *M* = 73.95%, *SD* = 24.81%), *t*(60) = 6.26, indicating that the effect of item difficulty on study selection is greater than item order.

Time allowed interacted with item order, *F*(1,118) = 13.44, $\eta_{p}^{2}$ = 0.10, *p* < 0.01. The simple effect analysis showed that time allowed was different for both the EMD group, *F*(1,118) = 564.16, *MSE* = 12936.02, *p* < 0.01, and the DME group, *F*(1,118) = 232.85, *MSE* = 5339.27, *p* < 0.01, indicating that the time allowed affected the order of study choice. Time allowed interacted with item difficulty, *F*(1,118) = 11.05, $\eta_{p}^{2}$ = 0.09 *p* < 0.01. The simple effect analysis showed a significant main effect for item difficulty in the 5-s condition, *F*(1,118) = 14.27, *MSE* = 120.42, *p* < 0.01 and in the no time constraint condition, *F*(1,118) = 36.53, *MSE* = 355.27, *p* < 0.01. For the different time limits (5 s, no limit), the easy items were prioritized during selection (EMD, *M* = 94.79%, *SD* = 6.94%), compared with difficult items (DME, *M* = 15.00%, *SD* = 19.31%), *t*(60) = 30.11. This indicated that the effect of item difficulty on item selection was greater than item order, so the influence of agenda was greater than habitual responding on study time allocation.

An ANOVA of third choices revealed a significant main effect of item difficulty, *F*(1,118) = 58.33, $\eta_{p}^{2}$ = 0.33, *p* < 0.01, and a significant main effect of time allowed, *F*(1,118) = 28.55, $\eta_{p}^{2}$ = 0.20, *p* < 0.01. Item difficulty interacted with item order, *F*(1,118) = 603.34, $\eta_{p}^{2}$ = 0.85, *p* < 0.01. The simple effect analysis showed that item order was different for easy items, *F*(1,118) = 7.00, *MSE* = 16.02, *p* < 0.01, but not for difficult items, indicating that the effect of item order on easy items is greater than on difficult items. Time allowed interacted with item order, *F*(1,118) = 7.9, $\eta_{p}^{2}$ = 0.06, *p* < 0.01. The simple effect analysis showed a significant main effect for item order in the 5-s condition, *F*(1,118) = 442.59, *MSE* = 5890.50, *p* < 0.01 and in the no time constraint condition, *F*(1,118) = 622.09, *MSE* = 7150.42, *p* < 0.01. This indicated that item order had effect on study selection is in different time constraints.

#### Self-paced Study

Mean self-paced study times are presented in **Table 3.**

**Table 3:** Self-paced study time in seconds for Experiment 2 (Ms, SDs).

Item order	Short time constraints (5 s)				No time constraint		
			
	Easy	Moderate	Difficult		Easy	Moderate	Difficult
EMD	1.29	1.40	1.16		6.39	9.63	11.94
	*SD* = 0.21	*SD* = 0.42	*SD* = 0.39		*SD* = 2.07	*SD* = 4.02	*SD* = 3.92
DME	1.03	2.06	1.38		8.11	13.99	11.31
	*SD* = 0.28	*SD* = 0.36	*SD* = 0.25		*SD* = 3.51	*SD* = 5.26	*SD* = 5.30


*EMD, easy items on the top of the array and difficult items on the bottom; DME, difficult items on the top of the array and easy items on the bottom.*

An ANOVA revealed a significant main effect of item difficulty, *F*(2,117) = 111.56, $\eta_{p}^{2}$ = 0.66, *p* < 0.01, a significant main effect of time allowed, *F*(1,118) = 1313.22, $\eta_{p}^{2}$ = 0.92, *p* < 0.01. Item difficulty interacted with item order, *F*(2,117) = 36.48, $\eta_{p}^{2}$ = 0.38, *p* < 0.01. The simple effect analysis showed that item order was different for both easy items, *F*(1,118) = 22.32, *MSE* = 2.46, *p* < 0.01, and difficult items, *F*(1,118) = 8.22, *MSE* = 208.24, *p* < 0.01, indicating that item order affects self-paced study. The descriptive statistics showed that participants spent more time on DME (*M* = 6.31) than on EMD (*M* = 5.30). Time allowed interacted with item difficulty, *F*(2,117) = 93.82, $\eta_{p}^{2}$ = 0.62, *p* < 0.01. The simple effect analysis showed that time allowed was different for both the short time limit condition (5s), *F*(2,236) = 610.17, *MSE* = 4487.86, *p* < 0.01, and no time limit condition, *F*(1,118) = 308.29, *MSE* = 2952.42, *p* < 0.01.

#### Recall Performance

We examined recall performance to determine the effect of study time allocation. The mean test accuracies are presented in **Table 4.**

**Table 4:** Test accuracies for Experiment 2 (Ms, SDs).

Item order	Short time constraints (5 s)				No time constraint		
			
	Easy	Moderate	Difficult		Easy	Moderate	Difficult
EMD	0.35	0.23	0.02		0.71	0.52	0.19
	*SD* = 0.15	*SD* = 0.16	*SD* = 0.05		*SD* = 0.14	*SD* = 0.17	*SD* = 0.18
DME	0.43	0.26	0.06		0.71	0.56	0.23
	*SD* = 0.11	*SD* = 0.10	*SD* = 0.07		*SD* = 0.16	*SD* = 0.14	*SD* = 0.16


*EMD, easy items in the top of the array and difficult items in the bottom; DME, difficult items in the top of the array and easy items in the bottom.*

An ANOVA revealed a significant main effect of item difficulty, *F*(2,236) = 12.90, $\eta_{p}^{2}$ = 0.10, *p* < 0.01, a significant main effect of time allowed, *F*(1,118) = 61.00, $\eta_{p}^{2}$ = 0.34, *p* < 0.01, and a significant main effect of item order, *F*(1,118) = 6.92, $\eta_{p}^{2}$ = 0.06, *p* < 0.05. Time allowed interacted with item difficulty, *F*(1,118) = 853.03, *MSE* = 504.31, $\eta_{p}^{2}$ = 0.87, *p* < 0.01, indicating that the item difficulty and time limit jointly affect the recall performance.

## Experiment 3

In order to verify the experimental results of Dunlosky and Ariel and compare them with results in junior high school students, we chose young adults as the participants to repeat the experimental procedure in Experiments 1 and 2.

### Experiment 3a

#### Method

Participants were 120 undergraduates (50% men and 50% women; mean age = 20.44 years, *SD* = 1.53) in Henan University. The materials, design, and procedure in this experiment was same as the Experiment 1.

#### Results and Discussion

##### Item selection

**FIGURE 3:**
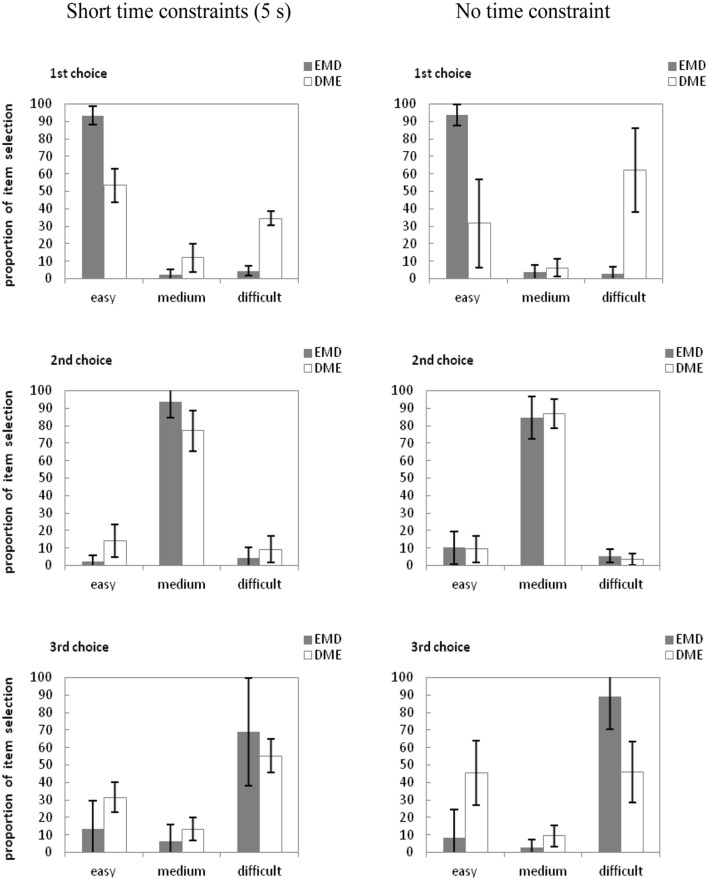
**Mean proportion of items selected for the first, second, and third choice across the word pair arrays in Experiment 3a.** E, easy word pair; M, moderately difficult word pair; D, difficult word pair. Error bars are standard errors of each mean.

The proportion of item selection in different difficulty and time constraints are presented in **Figure 3.**

We conducted a separate 2 (Item Order: EMD vs. DME) × 2 (Time Limit: 5 s vs. unlimited) × 2 (Item Difficulty: easy or difficult) analysis of variance (ANOVA) as in Experiment 1. The ANOVA for first choices revealed a significant main effect of item difficulty, *F*(1,118) = 9.76, $\eta_{p}^{2}$ = 0.08, *p* < 0.01. The main effect of time allowed was significant, *F*(1,118) = 533.28, $\eta_{p}^{2}$ = 0.81, *p* < 0.01. The main effect of item order also significant, *F*(1,118) = 58.54, $\eta_{p}^{2}$ = 0.33, *p* < 0.01. Item difficulty interacted with item order, *F*(2,236) = 30.31, $\eta_{p}^{2}$ = 0.20, *p* < 0.001. Time allowed interacted with item order, *F*(1,118) = 691.16, $\eta_{p}^{2}$ = 0.85, *p* < 0.01. Time allowed interacted with item difficulty, *F*(1,118) = 16.030, *MSE* = 318.304, $\eta_{p}^{2}$ = 0.82, *p* < 0.001. An ANOVA of third choices revealed a significant main effect of item difficulty, *F*(1,118) = 37.32, $\eta_{p}^{2}$ = 0.24, *p* < 0.001. And a significant main effect of time allowed, *F*(1,118) = 360.61, $\eta_{p}^{2}$ = 0.75, *p* < 0.001. Item difficulty interacted with item order, *F*(1,118) = 9.04, $\eta_{p}^{2}$ = 0.07, *p* < 0.01. This result showed that in line with the junior school students, the item order had decrease the effect of item difficulty in item selection and when the more time people had, the more trend to choose the item in item order happened.

##### Self-paced study

Mean self-paced study times are presented in **Table 5.**

**Table 5:** Self-paced study time in seconds for Experiment 3a (Ms, SDs).

Item order	Short time constraints (5 s)				No time constraint		
			
	Easy	Moderate	Difficult		Easy	Moderate	Difficult
EMD	1.52	1.42	1.59		3.87	6.64	10.18
	*SD* = 0.11	*SD* = 0.09	*SD* = 0.14		*SD* = 0.42	*SD* = 0.92	*SD* = 1.08
DME	1.48	1.39	1.62		3.72	6.57	10.23
	*SD* = 0.10	*SD* = 0.11	*SD* = 0.08		*SD* = 0.58	*SD* = 1.04	*SD* = 1.16


*EMD, easy items on the left of the array and difficult items on the right; DME, difficult items on the left of the array and easy items on the right.*

An ANOVA revealed a significant main effect of item difficulty, *F*(1,118) = 7857.80, $\eta_{p}^{2}$ = 0.98, *p* < 0.01, a significant main effect of time allowed, *F*(1,118) = 1928.36, $\eta_{p}^{2}$ = 0.94, *p* < 0.01. Time allowed interacted with item difficulty, *F*(1,118) = 593.887, $\eta_{p}^{2}$ = 0.83, *p* < 0.01. Different from the results in Experiment 1, no significant main effect of item order, and there was no interaction between the item difficulty and item order. This results showed that the item order had no effect on the adults’ self-paced study.

##### Recall performance

We examined recall performance to determine the effect of study time allocation. The mean test accuracies are presented in **Table 6.**

**Table 6:** Test accuracies for Experiment 3a (Ms, SDs).

Item order	Short time constraints (5 s)				No time constraint		
			
	Easy	Moderate	Difficult		Easy	Moderate	Difficult
EMD	0.41	0.18	0.03		0.83	0.61	0.27
	*SD* = 0.66	*SD* = 0.53	*SD* = 0.07		*SD* = 0.06	*SD* = 0.18	*SD* = 0.07
DME	0.42	0.21	0.04		0.79	0.57	0.28
	*SD* = 0.10	*SD* = 0.07	*SD* = 0.06		*SD* = 0.10	*SD* = 0.14	*SD* = 0.12


*EMD, easy items in the left of the array and difficult items in the right; DME, difficult items in the left of the array and easy items in the right.*

An ANOVA revealed a significant main effect of item difficulty, *F*(1,118) = 115.169, $\eta_{p}^{2}$ = 0.49, *p* < 0.01, and a significant main effect of time allowed, *F*(1,118) = 157.57, $\eta_{p}^{2}$ = 0.57, *p* < 0.01. No significant main effect of item order. Time allowed interact with item difficulty, *F*(1,118) = 2482.99, $\eta_{p}^{2}$ = 0.95, *p* < 0.01. The results indicated that item difficulty and time constraint jointly affect recall performance, and that item order has no effect on recall performance.

### Experiment 3b

#### Method

Participants were 120 undergraduates (50% men and 50% women; mean age = 19.58 years, *SD* = 1.05) in Henan University. The materials, design, and procedure in this experiment was same as the Experiment 2.

#### Results and Discussion

##### Item selection

**FIGURE 4:**
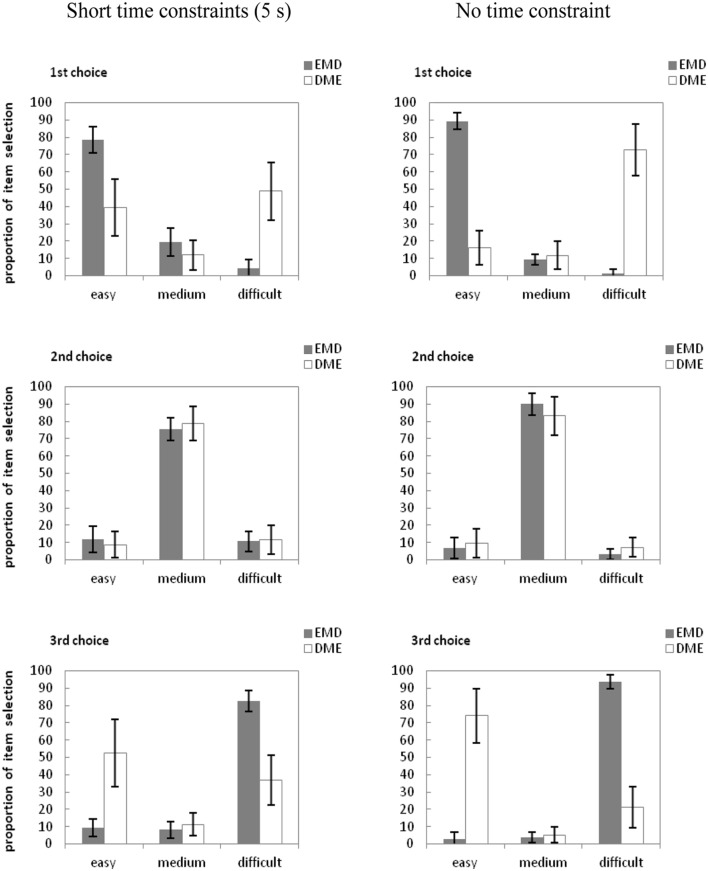
**Mean proportion of items selected for the first, second, and third choice across the word pair arrays in Experiment 3b.** E, easy word pair; M, moderately difficult word pair; D, difficult word pair. Error bars are standard errors of each mean.

The proportion of item selection in different difficulty and time constraints are presented in **Figure 4**

We conducted a separate 2 (Item Order: EMD vs. DME) × 2 (Time Limit: 5 s vs. unlimited) × 2 (Item Difficulty: easy or difficult) analysis of variance (ANOVA) as in Experiment 2. The ANOVA for first choices revealed a significant main effect of item difficulty, *F*(1,118) = 16.09, $\eta_{p}^{2}$ = 0.12, *p* < 0.01. The main effect of time allowed was significant, *F*(1,118) = 338.63, $\eta_{p}^{2}$ = 0.74, *p* < 0.01. Item difficulty interacted with item order, *F*(1,118) = 12.18, $\eta_{p}^{2}$ = 0.09, *p* < 0.01. Time allowed interacted with item order, *F*(1,118) = 691.16, $\eta_{p}^{2}$ = 0.85, *p* < 0.01. Time allowed interacted with item difficulty, *F*(1,118) = 1587.89, $\eta_{p}^{2}$ = 0.91, *p* < 0.01. An ANOVA of third choices revealed a significant main effect of item difficulty, *F*(1,118) = 60.80, $\eta_{p}^{2}$ = 0.34, *p* < 0.001. And a significant main effect of time allowed, *F*(1,118) = 269.16, $\eta_{p}^{2}$ = 0.69, *p* < 0.001. This result extended the finding of Experiment 2 that no matter the junior school students or the adults, even though the item order can affect their item selection, they also intend to select easy item as their first selection. But if they had the enough time to study, they prefer to choose items according to item order. And we can find from the figure that comparing with items which were presented from left to right (in Experiments 1 and 3a), the intend to chose items in item order was more stronger.

##### Self-paced study

Mean self-paced study times are presented in **Table 7.**

**Table 7:** Self-paced study time in seconds for Experiment 3b (Ms, SDs).

Item order	Short time constraints (5 s)				No time constraint		
			
	Easy	Moderate	Difficult		Easy	Moderate	Difficult
EMD	1.37	1.43	1.52		4.35	6.86	10.74
	*SD* = 0.12	*SD* = 0.09	*SD* = 0.19		*SD* = 0.66	*SD* = 0.58	*SD* = 0.95
DME	1.41	1.45	1.50		4.01	6.52	10.99
	*SD* = 0.12	*SD* = 0.08	*SD* = 0.15		*SD* = 0.72	*SD* = 0.69	*SD* = 0.98


*EMD, easy items on the top of the array and difficult items on the bottom; DME, difficult items on the top of the array and easy items on the bottom.*

An ANOVA revealed a significant main effect of item difficulty, *F*(1,118) = 14141.50, $\eta_{p}^{2}$ = 0.99, *p* < 0.01, a significant main effect of time allowed, *F*(1,118) = 3838.79, $\eta_{p}^{2}$ = 0.97, *p* < 0.01. Time allowed interacted with item difficulty, *F*(1,118) = 754.08, $\eta_{p}^{2}$ = 0.86, *p* < 0.01. Different from the results in Experiment 2, no significant main effect of item order. This results also showed that the item order had no effect on the adults’ self-paced study and when the time wasn’t enough, the effect of item difficulty on adults’ self-paced study would decrease.

##### Recall performance

We examined recall performance to determine the effect of study time allocation. The mean test accuracies are presented in **Table 8.**

**Table 8:** Test accuracies for Experiment 3b (Ms, SDs).

Item order	Short time constraints (5 s)				No time constraint		
			
	Easy	Moderate	Difficult		Easy	Moderate	Difficult
EMD	0.45	0.27	0.03		0.84	0.53	0.31
	*SD* = 0.80	*SD* = 0.57	*SD* = 0.43		*SD* = 0.80	*SD* = 0.88	*SD* = 0.94
DME	0.44	0.29	0.02		0.78	0.51	0.33
	*SD* = 0.76	*SD* = 0.70	*SD* = 0.42		*SD* = 0.16	*SD* = 0.86	*SD* = 0.81


*EMD, easy items in the top of the array and difficult items in the bottom; DME, difficult items in the top of the array and easy items in the bottom.*

An ANOVA revealed a significant main effect of item difficulty, *F*(1,118) = 26.45, $\eta_{p}^{2}$ = 0.18, *p* < 0.01, and a significant main effect of time allowed, *F*(1,118) = 515.44, $\eta_{p}^{2}$ = 0.81, *p* < 0.01. No significant main effect of item order. Time allowed interacted with item difficulty, *F*(1,118) = 2125.83, $\eta_{p}^{2}$ = 0.95, *p* < 0.01. And different with Experiment 2, Time allowed didn’t interact with item difficulty. The results is in line with previous findings on adults (Dunlosky and Ariel, 2011b) that item difficulty and time constraint jointly affect recall performance, and that item order has no effect on recall performance.

## General Discussion

### The Influences of Habitual Responding, Item Difficulty, and Time Constraints

The results showed that the item order greatly influenced item selection. The Chinese junior school students and young adults all tended to choose the items that were in the most prominent position (e.g., left or top position of the array) first, and they chose the items that were in the least prominent position (e.g., right or bottom position of the array) last. These findings are consistent with the observations of Dunlosky and Ariel (2011b) and Xie et al. (2014). This trend is more obvious when the material is structured vertically (top–bottom) compared to horizontally (left–right). Dunlosky and Ariel (2011b) interpreted their results as showing that the effect of reading habit (the reading order of one’s language), has the greatest influence on item selection. In order to verify this interpretation, they used the same experimental procedure to test native Arabic speakers who read from right to left. The results showed that participants’ decisions were biased by the direction in which they read text in their native language.

Item difficulty can also influence the order of study. Our findings show that more difficult material is less likely to be selected first and more likely to be selected last. This is consistent with the RPL framework (Metcalfe, 2002), which indicates that participants always spend more time on the easiest unlearned material and then gradually shift toward more difficult material. In our study, both item difficulty and item order contributed to item selection. Compared with difficult items, easy items were prioritized during selection for the different times allowed (5 s, unlimited) when they were in the same item order. Therefore, item difficulty had a greater influence on study selection than item order, and agenda influenced study time allocation more than habitual responding. These findings echo those of many previous studies indicating that participants prefer to choose easy items first (Son and Metcalfe, 2000; Metcalfe, 2002; Metcalfe and Kornell, 2005)

Our findings showed different choice tendencies for the different times allowed. Participants tended to choose easy items first when the time allowed was short (5 s) and to choose items based on habitual responding when the time allowed was long (no time constraint). This suggests that in order to achieve better recall performance and mastery of the items, junior school students tend to build agendas that involve choosing easy items first, based on the RPL. However, when the time allowed was long, students had enough time to master all the items, so the establishment of the agenda became less important. Then they tended to select items based on reading habit.

In terms of self-paced study, experiments on junior school students showed that item order affected the time spent on items of different difficulty. Participants spent more time on the DME group than on the EMD group, and time allowed interacted with item order when materials were presented in a vertical structure. The results indicated that vertical structure had a greater effect on self-paced study than horizontal structure. In contrast, in Experiment 3 and previous work (Dunlosky and Ariel, 2011b; Xie et al., 2014) which with young adult participants did not show a significant effect of item order and habitual responding on self-paced study. Whether the materials were presented in a horizontal or vertical structure had no significant effect on the self-paced study of adults. For junior school students, the item difficulty also interacted with item order in terms of self-paced study time. When the materials were presented from left to right in DME, the time spent on moderately difficult items and difficult items did not differ in Experiment 1. However, when the materials were presented in a vertical structure, junior school students spent more time on moderately difficult items than on difficult items. This pattern differs from findings with young adults in Experiment 3 and previous work (Son and Metcalfe, 2000; Dunlosky and Ariel, 2011b; Xie et al., 2014), whose self-paced study is not affected by item order and who always spend the most time on difficult items.

Regarding the difference between junior school students and young adults, our explanation is that junior school students have a larger extraneous cognitive load than young adults when the materials are presented in a vertical structure and the junior school students have a lower capacity to inhibit the irrelevant information. So the habitual responses had a larger influence on junior school students’self-paced study. In an ABR framework, the process of agenda construction and execution was a metacognitive process and needed working memory capacity (Dunlosky and Ariel, 2011a, pp. 108–120). Research on working memory across the life span showed that the young adults had higher working memory capacity and greater ability of inhibition of irrelevant information than junior school students had (Swanson, 1999; Zhang and Liu, 2006). Further, the cognitive load and working memory are closely related (Barrouillet et al., 2007). According to the cognitive load theory, the process of memory can be affected by the extraneous cognitive load. Extraneous cognitive load is associated with the presentation of learning materials and is independent of the nature of the learning material (van Merriënboer and Ayres, 2005), so the order of materials (horizontal or vertical structure) relates to the extraneous cognitive load. Many researches have shown that when stimulus and response sets vary along horizontal and vertical dimensions, the horizontal dimension is dominant (Nicoletti and Umilta, 1985; Nicoletti et al., 1988; Vu et al., 2000), and people require more cognitive resources to code the stimulus in the vertical dimension (Heister et al., 1990; Vu and Proctor, 2001). The presentation of learning materials belonged to the cognitive load, as did information from the students’ environment. Dunlosky and Ariel (2011b) explained that “the central executive receives information from the memory system (monitoring), and it can use this information to change the state of this system (control), such as by focusing attention on currently unactivated information in the environment.” Their study also showed that habitual responses will influence study-time allocation and can undermine ABR, and these habitual responses are expected to have a larger influence when the capacity of the central executive is exceeded.

The junior school students varied their self-paced study time according to the different times allowed. When the time allowed was short (5 s), they spent the least amount of time on easy items and the most time on moderately difficult items. Differently, young adults allocate nearly equal amounts of time to all items when the time allowed is short (Metcalfe and Kornell, 2003; Dunlosky and Ariel, 2011b; Xie et al., 2014). Metcalfe and Kornell (2003) found that best performance resulted when most time was given to the medium-difficulty items. This suggests that junior school students tend to choose the most effective strategy in short time constraints. Compared with the recall performance in the condition in which they did not spend most time on moderate difficulty items (EMD group in experiment, *M* = 0.19), the recall performance in rest conditions had better results (DME group in Experiment 1, *M* = 0.25; EMD group in Experiment 2, *M* = 0.23; DME group in Experiment 2, *M* = 0.26). This result verified the effectiveness of this strategy. Although young adults did not adopt this strategy, they still had better recall performance than junior school students in Experiment 3 had. This showed that the capacity of metacognition had a maximum impact on study time allocation. When there was no time limit, the junior school students spent most time on difficult items and least time on easy items in EMD groups. But in DME group, they spent most time on moderate items and least time on easy items. Differently, young adults significantly vary the time spent on items of different levels of difficulty, with difficult > moderately difficult > easy items. Once again, these results showed that the junior school students’ study time allocation was more likely to be affected by the presentation of learning materials than that of the young adults.

In terms of recall performance, previous studies on young adults and Experiment 3 both indicate that item order has no influence on test accuracy (Dunlosky and Ariel, 2011b; Xie et al., 2014). In contrast, our findings show that when the materials are presented in the time allowed is short (5 s), item order affects the recall performance of junior school students. It was because junior school students spent more time on these materials, and their behavior of spending more time was affected by their habitual response. The results of self-paced study showed that junior school students spent more time on moderately difficult items in the DME group than in the EMD group in short time constraints, and the recall performance of moderate difficulty items in the DME group was better than performance on those items in the EMD group. This suggested that habitual responses could influence the recall performance by influencing the self-paced study of junior school students.

### The Relationship between Agenda and Habitual Responding

This study verified the existence of agenda construction and habitual responding in study time allocation, as well as differences in study time allocation with different time constraints. The results showed that junior school students can build and execute an agenda based on RPL in a short time limit condition (5 s), and they tend to select the items based on habitual responding when the time limit is long. Our findings suggest that the agenda and habitual responding had a joint effect on native Chinese speakers’ study time allocation. When learners were able to build and execute an agenda, their time allocation was mainly affected by the agenda, but if they did not have to build and execute the agenda, they always allocated their time based on habitual responding.

Dunlosky and Ariel (2011b) results suggest that even when learners have developed an agenda, the process of executing can be affected by habitual responding. In the present study, when the difficult items were presented in the most prominent position (left or top), they were prioritized during selection, compared with easy items presented in the least prominent position (right or bottom). This result indicates that item order affects the order of study. Therefore, we have verified that although the development and execution of agendas weakens habitual responding to a certain extent, the process of executing the agenda can also be influenced by habitual responding. However, the effect of students’ habitual responding on self-paced study time and recall performance is reduced greatly. This indicates that the development and execution of an agenda more strongly determines study time allocation and that item order does not affect all aspects of study time allocation.

## Conclusion

In the present experiments, we systematically investigated the effects of item difficulty, item order, and time limitation on study time allocation of junior school students and compared the study time allocation of junior school students and younger adults. We also verified the relationship between agenda and habitual responses. As expected, agenda and habitual responding have a combined effect on study time allocation, and the contribution of agenda is greater than that of habitual responding. Although we found that the effect of habitual responding on self-paced study and recall performance of junior school students is greater than its effect on young adults, their study time allocation is more likely to be affected by external conditions. This result should be verified in more experiments with participants from different cultural backgrounds. Although the junior school students chose the most effective strategy (giving most time to the medium-difficulty items) in short time constraints, the young adults’ recall performance still was better than theirs. This indicated that age differences and the individual learning capacity affect learning greatly. Whether the junior middle school students always choose this strategy should also be verified in future research.

## Author Contributions

FW is a graduate student at the Southwest University of Department of psychology for Doctor’s degree and research in personality and organizational culture. In the study period of Master, her research interests include memory, study time allocation, and metacognition of students. YJ is a professor at the Henan University for research on students education. Her academic focus include students’ memory, metacognition, study time allocation, level of aspiration, aggressive behavior. QQ is a professor at the Southwest University for Research on organizational psychology. His academic focus include Organization image, public relations, social etiquette, interpersonal communication, social psychology, mental health, implementation management, and emergency prevention and response.

## Conflict of Interest Statement

The authors declare that the research was conducted in the absence of any commercial or financial relationships that could be construed as a potential conflict of interest.
